# No signal of deleterious mutation accumulation in conserved gene sequences of extant asexual hexapods

**DOI:** 10.1038/s41598-019-41821-x

**Published:** 2019-03-29

**Authors:** Alexander Brandt, Jens Bast, Stefan Scheu, Karen Meusemann, Alexander Donath, Kai Schütte, Ryuichiro Machida, Ken Kraaijeveld

**Affiliations:** 1 University of Göttingen, JF Blumenbach Institute of Zoology and Anthropology, Untere Karspüle 2, D-37073 Göttingen, Germany; 2 University of Lausanne, Department of Ecology and Evolution, UNIL Sorge, Le Biophore, CH-1015 Lausanne, Switzerland; 3 University of Freiburg, Biology I, Evolutionary Biology & Ecology, Hauptstraße 1, D-79104 Freiburg, Germany; 4 Center for Molecular Biodiversity Research (ZMB), Zoological Research Museum Alexander König, Adenauerallee 160, D-53113 Bonn, Germany; 5 University of Hamburg, Faculty of Mathematics, Informatics and Natural Sciences, Department of Biology, Institute of Zoology, Research Unit Animal Ecology and Conservation, Martin-Luther-King-Platz 3, D-20146 Hamburg, Germany; 6 Sugadaira Research Station, Mountain Science Center, University of Tsukuba, 1278-294, Sugadaira Kogen, Ueda, Nagano, 386-2204 Japan; 7 University of Amsterdam, Institute for Biodiversity and Ecosystem Dynamics, Science Park 904, 1090 GE Amsterdam, The Netherlands

## Abstract

Loss of sex and recombination is generally assumed to impede the effectiveness of purifying selection and to result in the accumulation of slightly deleterious mutations. Empirical evidence for this has come from several studies investigating mutational load in a small number of individual genes. However, recent whole transcriptome based studies have yielded inconsistent results, hence questioning the validity of the assumption of mutational meltdown in asexual populations. Here, we study the effectiveness of purifying selection in eight asexual hexapod lineages and their sexual relatives, as present in the 1 K Insect Transcriptome Evolution (1KITE) project, covering eight hexapod groups. We analyse the accumulation of slightly deleterious nonsynonymous and synonymous point mutations in 99 single copy orthologue protein-coding loci shared among the investigated taxa. While accumulation rates of nonsynonymous mutations differed between genes and hexapod groups, we found no effect of reproductive mode on the effectiveness of purifying selection acting at nonsynonymous and synonymous sites. Although the setup of this study does not fully rule out nondetection of subtle effects, our data does not support the established consensus of asexual lineages undergoing ‘mutational meltdown’.

## Introduction

The ubiquitous prevalence of sex among eukaryotes is surprising given that sexual reproduction involves manifold evolutionary costs as compared to obligate asexuality^1–3^. One prediction for the benefit of sex in the long-term is the increased effectiveness of purifying selection^4^. The rationale is that segregation, recombination and outcrossing enable the uncoupling of linked loci with different selection coefficients, such that selection can act on different loci independently^5^. This accelerates adaptation and the purging of slightly deleterious mutations and facilitates the restoration of least loaded genotypes that are continuously lost by drift^5–8^. Asexual lineages lack these benefits and are therefore predicted to succumb to ‘mutational meltdown’^9^.

A multitude of studies have tested the prediction of impeded effectiveness of purifying selection (i.e. selective removal of deleterious mutations) in non-recombining genomic regions, such as mitochondria or (neo-) Y chromosomes as well as different lineages of asexual eukaryotes^10–13^. Their results have led to the established consensus that slightly deleterious mutations accumulate in the absence of sex. However, many of the studies that have investigated purifying selection in asexual species were based on only few individual genes and recent studies based on whole transcriptome comparisons between asexual and related sexual lineages did not find consistent support: while accumulation of slightly deleterious mutations was found in asexual *Timema* stick insects, *Oenothera* evening primroses and *Boechera* rockcress, it was absent in *Lineus* ribbon worms as well as four aphid species and, opposite to predictions, reduced in asexual as compared to sexual oribatid mites^14–19^. Moreover, all whole transcriptome based studies found excessive variation among genes, and hence doubts have been raised about the robustness of inferences drawn from single gene analyses^20^. These conflicting results highlight the need for more studies of larger gene sets along with broader taxonomic sampling to infer whether or not accumulation of deleterious mutations is indeed a consequence of asexual reproduction.

Here, we study the effectiveness of purifying selection in obligately asexual and related sexual hexapod lineages covering eight hexapod groups using transcriptome data generated by the 1KITE project (1 K Insect Transcriptome Evolution, www.1kite.org). As parthenogenesis is a lineage-level trait, we use the term ‘lineage’, whenever to distinguish between the two reproductive modes (sexual and asexual) within a hexapod group, from here on. With the term ‘hexapod group’ we refer to one of the following analysed taxa: Collembola, Zygentoma, Phasmatodea, Mantodea, Thysanoptera, Sternorrhyncha, Hymenoptera and Psocodea, respectively.

**Figure 1:**
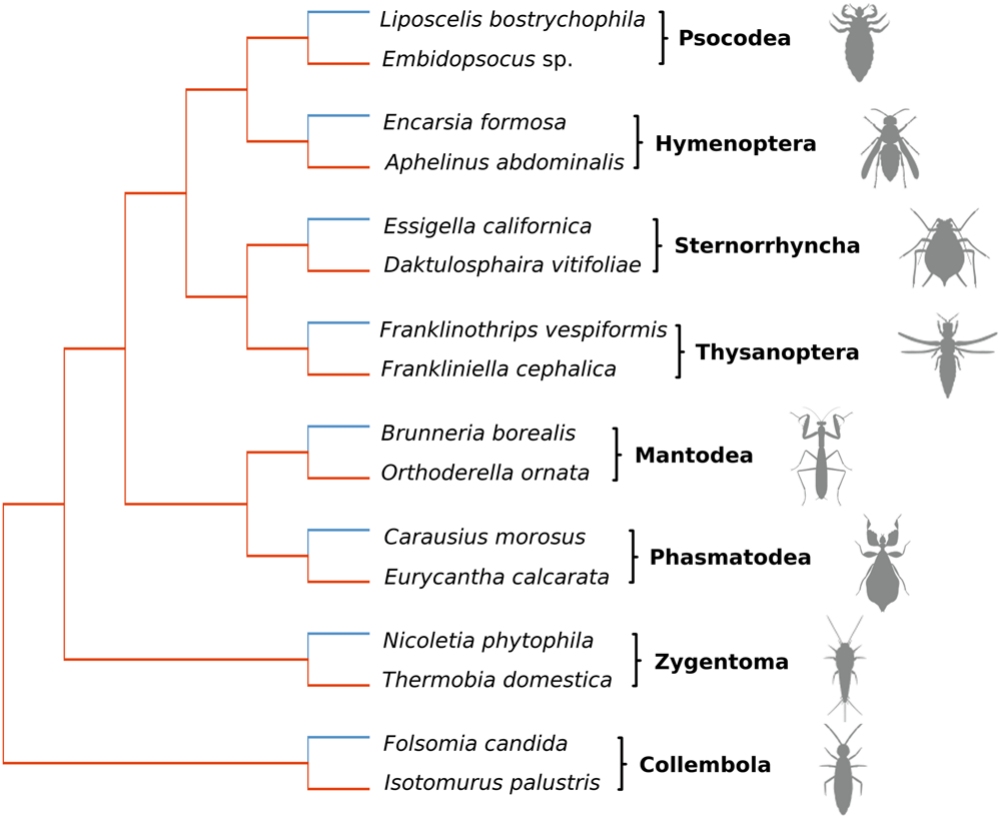
Cladogram of 16 hexapod species analysed in this study. The cladogram was manually built based on the phylogeny published by Misof *et al*. 2014 (see Methods)^33^. The taxon sampling includes eight asexual lineages along with their closest sexual relatives as present in 1KITE, covering eight hexapod groups. Sexual and asexual lineages are depicted in red and blue, respectively. Silhouettes courtesy of Hans Pohl.

We compared the accumulation of slightly deleterious mutations in nuclear protein-coding genes between eight asexual lineages and their sexual relatives as available from 1KITE (see Fig. 1). To this end, we first inferred divergence at nonsynonymous sites normalised for background mutation rates (dN/dS), and the potential ‘deleteriousness’ of nonsynonymous substitutions. Second, we investigated selection on Codon Usage Bias (CDC), because selection also acts at synonymous sites^21^. We based all analyses on 99 single copy orthologues which we found to be under purifying selection in the analysed lineages. We found extensive variation in dN/dS and CDC among genes and between hexapod groups, but no overall difference between reproductive modes.

## Methods

### Species selection

We searched for obligately asexual lineages within the 1KITE species list based on primarily van der Kooi & Schwander 2014^22^, Vershinina & Kuznetsova 2016^23^ and the Tree of Sex database^24^. We found eight hexapod groups with obligately asexual lineages represented by the following species: *Folsomia candida* (Collembola), *Nicoletia phytophila* (Zygentoma), *Carausius morosus* (Phasmatodea), *Brunneria borealis* (Mantodea), *Franklinothrips vespiformis* (Thysanoptera), *Essigella californica* (Sternorrhyncha), *Encarsia formosa* (Hymenoptera) and *Liposcelis bostrychophila* (Psocodea). In all analysed species, parthenogenesis is thelytokous. In *C. morosus* and *E. californica* it is obligately apomictic whereas in *F. candida*, *F. vespiformis* and *E. formosa* parthenogenesis is induced by bacteria of the genus *Wolbachia* and in *L. bostrychophila* by the genus *Ricketsia*^25–31^. We found no information concerning the mode of parthenogenesis in *B. borealis* and *N. phytophila* in the literature. Further, we selected per hexapod group the closest sexual relatives to the asexual lineages present in 1KITE, namely *Isotomurus palustris* (Collembola), *Thermobia domestica* (Zygentoma), *Eurycantha calcarata* (Phasmatodea), *Orthoderella ornata* (Mantodea), *Frankliniella cephalica* (Thysanoptera), *Daktulosphaira vitifoliae* (Sternorrhyncha), *Aphelinus abdominalis* (Hymenoptera) and *Embidopsocus* sp. (Psocodea). For information on first describers of species used, see Supplementary Table S1.

### Data acquisition

We downloaded published and most current transcriptome assemblies from Genbank and TSA of the following species: *A. abdominalis, E. formosa*, *E. californica*, *F. candida*, *F. cephalica*, *L. bostrychophila*, *T. domestica* and *Xibalbanus* cf. *tulumensis*^32–34^. For remaining species, we obtained unpublished transcriptome assemblies from the 1KITE consortium (RNA extraction, cDNA library generation, sequencing, assembly and contaminant removal was done as described in detail in Misof *et al*. 2014 and Peters *et al*. 2017)^33,35^. Assemblies were used as input for identification of orthologous sequences among all studied species. For information on References, BioSample IDs and Bioproject IDs of assembled transcriptomes, see Supplementary Table S1.

### Orthologue detection and alignment processing

To infer orthologue protein-coding genes among the 16 hexapod species, we used the Orthologous MAtrix (OMA) version 2.1.1 installed on the Vital-IT computing cluster^36^. To generate the input for OMA, we extracted long Open Reading Frames (ORFs) and predicted likely coding regions with Transdecoder version 2.0.1 using default options for each transcript and species^37^. Amino acid sequences of likely coding regions of each of the 16 species were passed to OMA together with an unrooted cladogram built by reducing a published phylogeny of hexapods to the eight hexapod groups analysed in this study^33^, which resulted in 286 orthologues shared among all analysed species. We aligned the amino acid sequences (ORFs) of each of the 286 orthologues with M-Coffee using a combination of different alignment methods creating a consensus alignment (option clustalw2_msa muscle_msa kalign_msa mafftgins_msa t_coffee_msa)^38^. Based on these, we generated corresponding codon alignments from the original nucleotide sequences with T-Coffee version 11.00.8^38^. As orthologue detection based on naturally incomplete datasets such as transcriptome data can lead to the detection of false positives, e.g. due to loci with paralogous sequences, we tested all protein sequences of the 286 orthologues of all 16 species for their presence in a precompiled set of orthologues of insects (insecta_hmmer3.1; www.deep-phylogeny.org/hamstr/download/datasets/hmmer3) using HaMStR version 13.2.6^39^. We only kept multiple sequence alignments of orthologues for which all species had an equivalent in the precompiled orthologue set leaving a set of 153 loci for further analyses. We curated these multiple sequence alignments using Gblocks version 0.91b with sequence type set to codons (*t* = *c*) and minimum block length set to 4 (*b*4 = *4*)^40^.

### Accumulation of nonsynonymous mutations

As a measure of purifying selection, we analysed the divergence at nonsynonymous sites normalised for background substitution rates (dN/dS) using CodeML as implemented in PAML version 4.9^41^. For this, we first manually constructed an unrooted cladogram of the 16 analysed species based on a published hexapod phylogeny^33^. To exclude orthologues that were under positive selection, we ran BUSTED as implemented in HYPHY version 2.3.10^42^ providing as input the unrooted cladogram described above and the multiple sequence alignments of the 153 orthologues. We found 54 loci showing signatures of episodes of positive selection, which left 99 loci for subsequent analyses (for GenBank Accession numbers, see Supplementary Table S2). CodeML relies on a Maximum Likelihood framework to estimate the goodness of fit of a codon substitution model to a sequence alignment and an unrooted species tree with gene-specific branch lengths for inference of branch-specific dN/dS ratios. We calculated per-gene branch lengths for the unrooted cladogram based on each of the 99 codon alignments, accordingly, using RAxML version 8.2.8^43^ with GTRGAMMAI (with four GAMMA rate categories) set as model of sequence evolution. We modified a custom script used by Brandt *et al*.^15^ (see Supplementary information) to pass the loci-specific branch lengths and fixed species tree together with each codon alignment to CodeML for divergence rate estimations. Due to the 1KITE taxon sampling, the asexual and sexual lineages of each hexapod group used in our analysis are likely not natural sister lineages (i.e. not the closest extant relatives) and therefore the time of transition to asexuality (or the split from the closest sexual relative) was unknown. As this could potentially lead to an overestimation of dN/dS under a model restricted to only two rates (one for asexual and one for sexual branches), we chose a free model allowing for different dN/dS ratios, one for each branch in the tree. For between-species comparisons of dN/dS ratios, we excluded all dN/dS ratios of internal branches and four terminal branch dN/dS ratios that were > 1, indicating positive selection acting at one branch, from statistical analyses. We then tested whether branch-specific dN/dS ratios differed according to (I) gene (II) reproductive mode, or (III) hexapod group using a permutation ANOVA with 5,000 bootstrap replicates (available at https://gist.github.com/KamilSJaron/358c997698b67486be47d4e8eef2921d)^44,45^. Differences in dN/dS can be driven by differing synonymous substitution rates and, in the long-term, different levels of saturation at synonymous sites. Given the old age of the splits between sexual and asexual lineages (~40 myo for Phasmatodea – ~160 myo for Zygentoma)^33,46^ we tested for differences in branch-specific dS as described above. To infer whether or not sexual and asexual lineages within individual hexapod groups differed in dN/dS ratios, we compared between reproductive modes using Wilcoxon signed-rank tests. All statistical analyses were done in R version 3.4.4^47^.

### ‘Deleteriousness’ of nonsynonymous mutations

To infer the ‘deleteriousness’ of nonsynonymous substitutions we analysed hydrophobicity changes from ancestral to replacement amino acids along the terminal branches of the phylogenetic tree. Hydrophobic interactions are the main determinants of the 3D conformation of proteins and thus an indicator of protein stability^48^. Inference of ancestral amino acids relies on the presence of an outgroup sequence included in the input amino acid alignments and the phylogenetic tree (cladogram with loci-specific branch lengths) used in analyses with CodeML. Therefore, we first searched for orthologues shared among all 16 hexapod species plus the crustacean *Xibalbanus* cf. *tulumensis* (previously *Speleonectes* cf. *tulumensis*), as a representative of Remipedia, the sister-group of hexapods^32,33^. For this, we predicted ORFs from the assembled transcriptome of *X.* cf. *tulumensis* and checked the ORFs of *X*. cf. *tulumensis* for presence of orthologues in the precompiled orthologue set of insects as described above. We found 73 ORFs of *X*. cf. *tulumensis*, each of them orthologous to one of the previously inferred 99 clusters of orthologues of the 16 hexapod species (for GenBank Accession numbers, see Supplementary Table S2). We again aligned the amino acid sequences (ORFs, including now the sequences of *X*. cf. *tulumensis*) of the 73 clusters of orthologues and subsequently generated corresponding codon alignments from the original nucleotide sequences as described above. Further, we added *X*. cf. *tulumensis* to the unrooted manually constructed cladogram used for the calculation of branch lengths for dN/dS ratio analyses as outgroup to all hexapods. Using this tree as a fixed topology, we calculated branch lengths for each orthologue locus individually as described above for analyses of nonsynonymous mutation accumulation, and then translated the curated alignments into amino acids using EMBOSS version 6.6.0^49^. To predict ancestral amino acid sequences for each internal node in each inferred tree from each gene, we passed the 73 amino acid codon alignments individually with its respective species trees (and estimated loci-specific branch lengths) to CodeML using the modified custom script mentioned above (see Supplementary information). We determined the strength of hydrophobicity changes (Hydrophobicity Scores; HS) for each of the amino acid transitions along the terminal branches of the species tree using a hydrophobicity scoring (HS) matrix^44,50^. HS indicates the ‘deleteriousness’ of a nonsynonymous mutation by measuring the strength in hydrophobicity change from ancestral to replacement amino acid. The lower the HS, the stronger is the change in hydrophobicity and, hence, the deleteriousness of the underlying nonsynonymous mutation. We compared values of HS between the two reproductive modes using Generalised Linear Mixed Models (GLMM) implemented in the R package lme4 with gene nested in species set as random effect, correction for overdispersion and Poisson distribution fitting^51^.

### Accumulation of synonymous mutations

Synonymous mutations are generally assumed to be neutral but can be subject to purifying selection because different codons can influence the speed and accuracy of translation^21^. Hence, we also analysed the effectiveness of selection acting on Codon Usage Bias (CUB). First, we inferred the existence of CUB for each of the 99 orthologues of the 16 species using the Effective Number of Codons (Nc) as measure with the software codonW version 1.4^52,53^. The Nc specifies the deviation of observed codon usage from equal usage of all codons ranging from 20 (each amino acid is encoded by one codon only; strong CUB) to 61 (equal use of all possible codons; no CUB). Next, we inferred selection on CUB using the Codon Deviation Coefficient (CDC)^54^. Measurement of CDC allows for cross-species comparisons by correcting for background nucleotide composition and is particularly robust because, unlike dN/dS ratio analyses, it does not rely on likelihood and branch length estimates. The CDC represents the deviation of expected CUB based on observed positional GC and purine contents from observed CUB, ranging from 0 (no deviation; no detectable selection on CUB) to 1 (maximum deviation; strong selection on CUB). We estimated CDC for the processed alignments using Composition Analysis Toolkit version 1.3^54^ and analysed it for an effect of I) gene, II) reproductive mode, and III) hexapod group and inferred within-group differences as described above for statistical analyses of dN/dS ratios.

## Results

### Accumulation of nonsynonymous mutations

**Figure 2:**
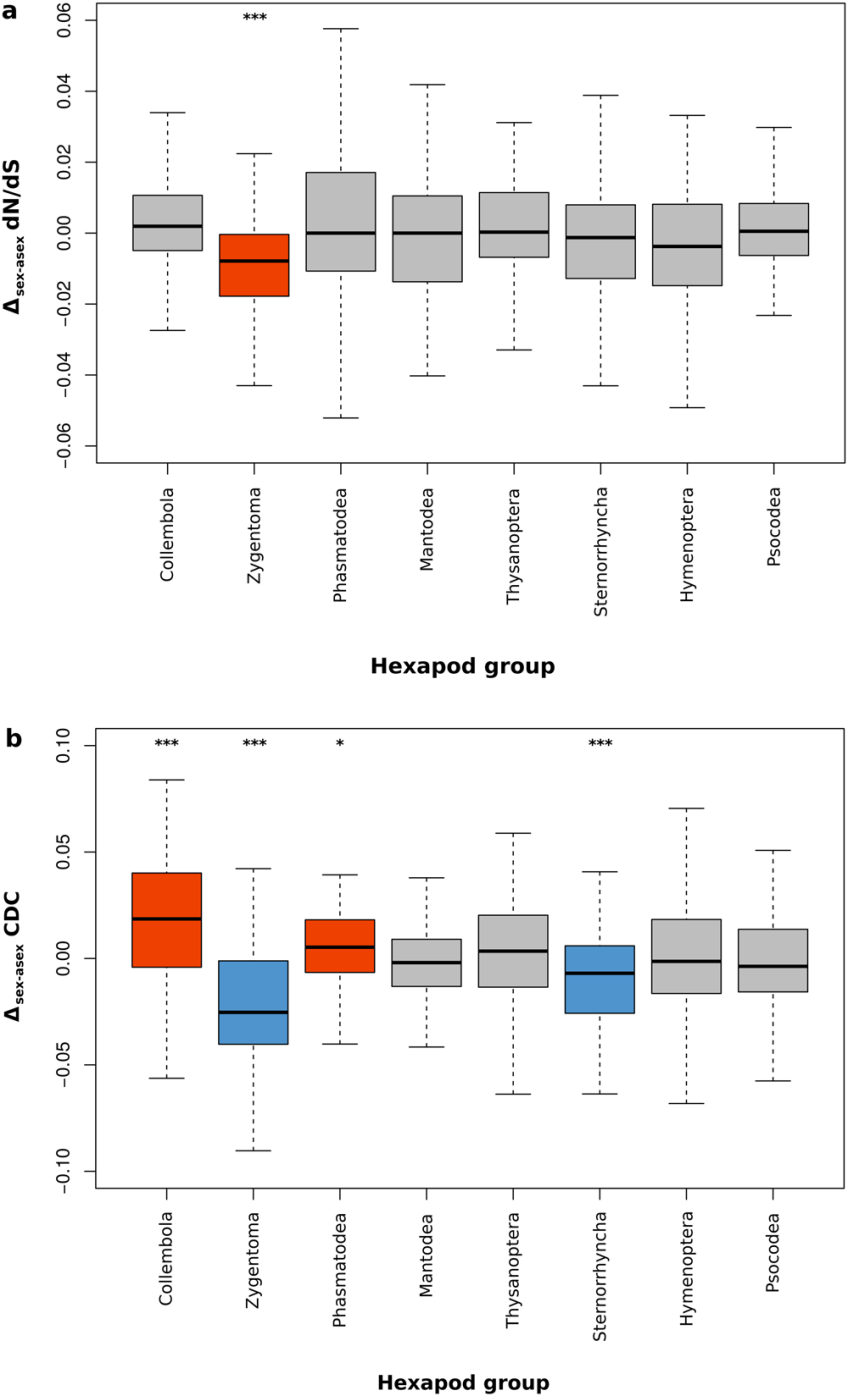
Per-gene differences in (**a**) dN/dS and (**b**) CDC between reproductive modes within each hexapod group. The boxplots show the distribution of per-gene differences in dN/dS between the sexual and asexual terminal branch (Δ_sex-asex_ dN/dS) and of per-gene differences in CDC between the sexual and asexual species (Δ_sex-asex_ CDC) of each of eight hexapod groups for 99 orthologues. For better representation, the ordinate is restricted to between −0.06 and 0.06 and −0.1 and 0.1, respectively, and outliers outside of 1.5 times the interquartile range (whiskers) are excluded. For dN/dS seven of eight within-hexapod-group comparisons between sexual and asexual branches yielded non-significant results (see Table 1). The red plot denotes a significantly lower per-gene dN/dS in sexual as compared to asexual terminal branches of Zygentoma (*V* = 3786, *P* < 0.001***; Wilcoxon signed-rank test). For CDC four of eight within-hexapod-group comparisons between sexual and asexual branches yielded significant results (see Table 1). There was significantly higher per-gene CDC in sexual as compared to asexual Collembola and Phasmatodea (red plots; *V* = 1191, *P* < 0.001*** and *V* = 1832, *P* = 0.025*, respectively; Wilcoxon signed-rank test) contrasting a significantly lower per-gene CDC in sexual as compared to asexual Zygentoma and Sternorrhyncha species (blue plots; *V* = 4198, *P* < 0.001 and *V* = 3562, *P* < 0.001, respectively; Wilcoxon signed-rank test).

We estimated nonsynonymous to synonymous divergence (dN/dS) along individual branches of a phylogenetic tree comprising eight asexual and eight sexual hexapod species for 99 single-copy orthologous protein-coding genes under purifying selection (see Fig. 1; Methods). In genes under purifying selection, nonsynonymous mutations have likely deleterious effects, hence, a higher dN/dS ratio indicates less effective purifying selection^55^. Consistent with the expectation for loci under purifying selection, dN/dS ratios at terminal branches were generally low (mean dN/dS = 0.032). Contrasting the established consensus on deleterious mutation accumulation in asexual lineages, there was no difference in dN/dS when compared between sexual and asexual branches over all hexapod groups (gene effect *P* < 0.001, reproductive mode effect *P* = 0.488, hexapod group effect *P* = 0.048, interaction reproductive mode * hexapod group *P* = 0.145; Permutation ANOVA). The absence of a difference between reproductive modes was not driven by a difference in dS (gene effect *P* < 0.001, reproductive mode effect *P* = 0.278, hexapod group effect *P* < 0.001, interaction reproductive mode * hexapod group *P* = 0.004; Permutation ANOVA). There was significant among-gene variation in dN/dS (range 0–0.454; variance 1.04 * 10^−3^) and a significant difference in dN/dS among hexapod groups. To detect whether or not the effectiveness of purifying selection differed between reproductive modes within each hexapod group, we compared dN/dS between reproductives modes in each hexapod group on a per-gene basis. For Zygentoma, we found significantly lower per-gene dN/dS in the sexual as compared to the asexual terminal branch indicating more effective purifying selection for the sexual branch (see Table 1; Fig. 2a red box).

**Table 1:** *V* and *P* values of within-hexapod group comparisons of dN/dS and CDC between reproductive modes. Values were inferred by comparing dN/dS and CDC per gene between the sexual and asexual lineages in each of eight hexapod groups using Wilcoxon signed-rank tests. Underlined and bold measures indicate more effective purifying selection in sexual species (underlined) and asexual species (bold), respectively (see Fig. 2a,b). Coll.: Collembola; Zyg.: Zygentoma; Phas.: Phasmatodea; Man.: Mantodea; Thys.: Thysanoptera; Stern.: Sternorrhyncha; Hym.: Hymenoptera; Psoc.: Psocodea.

Measure	Hexapod group							
	Coll.	Zyg.	Phas.	Man.	Thys.	Stern.	Hym.	Psoc.
dN/dS	*V* = 1886	*V* = 3786	*V* = 1747	*V* = 1263	*V* = 2008	*V* = 2382	*V* = 2845	*V* = 2056
	*P* = 0.107	*P* < 0.001	*P* = 0.381	*P* = 0.585	*P* = 0.314	*P* = 0.179	*P* = 0.092	*P* = 0.407
CDC	*V* = 1191	***V*** = **4198**	*V* = 1832	*V* = 2593	*V* = 2040	***V*** = **3562**	*V* = 2471	*V* = 2737
	*P* < 0.001	***P*** < **0.001**	*P* = 0.025	*P* = 0.682	*P* = 0.129	***P*** < **0.001**	*P* = 0.99	*P* = 0.361

### ‘Deleteriousness’ of nonsynonymous mutations

In addition to nonsynonymous mutation accumulation in asexual hexapod lineages, purifying selection is expected to lead to more deleterious amino acid transitions in asexual lineages as reflected by stronger hydrophobicity changes from ancestral to replacement amino acids along asexual and sexual terminal branches^56^. Contrasting this expectation, but consistent with our dN/dS estimates, HS were similar between asexual and sexual branches (*z* = −0.152; *P* = 0.879; GLMM; see Supplementary Fig. S1). Percentages of ancestral to replacement amino acid transitions with more dissimilar hydrophobicity (HS < 90) were similar between asexual and sexual branches (44.936% and 44.893% of all asexual and sexual transitions, respectively).

### Accumulation of synonymous mutations

We assessed whether or not purifying selection on synonymous sites was less effective in asexual as compared to sexual hexapod lineages by inferring selection on CUB. To infer whether or not the species investigated use some codons preferentially over others, we first analysed the Effective Number of Codons (Nc). Nc ranges from 20 (each amino acid is encoded by one codon only; strong CUB) to 61 (equal use of all possible codons; no CUB). Overall, all species showed CUB, with Nc means of species ranging from 40.6 in *D. vitifoliae* (Sternorrhyncha) to 54.1 in *F. candida* (Collembola; see Supplementary Fig. S2) which is comparable to values of Nc found in other invertebrate groups, e.g. bivalves and nematodes^57,58^. Afterwards, we directly inferred selection on CUB using CDC as a measure. CDC calculates the deviation of observed from predicted CUB by correcting for background nucleotide composition ranging from 0 (no selection on CUB) to 1 (effective selection on CUB). Consistent with the results from analyses of nonsynonymous mutation accumulation and ‘deleteriousness’ of nonsynonymous mutations, there was no difference in per-gene CDC between reproductive modes (gene effect *P* < 0.001, reproductive mode effect *P* = 0.283, hexapod group effect *P* < 0.001, interaction reproductive mode * hexapod group *P* < 0.001; Permutation ANOVA). Further, there was significant among gene variation for CDC (range 0.064–0.329; variance 1.653 * 10^−3^) and a significant difference between hexapod groups but, unlike for dN/dS estimates, there was also a significant interaction between hexapod group and reproductive mode. As for dN/dS, we compared per-gene CDC between reproductive modes in each hexapod group, individually. Four of eight within-hexapod-group comparisons between sexual and asexual species yielded significant results (see Table 1; Fig. 2b red and blue boxes, respectively). For Collembola and Phasmatodea, there was a significantly higher per-gene CDC in sexual as compared to asexual species indicating more effective selection on CUB in sexual species, whereas for Zygentoma and Sternorrhyncha there was a significantly lower per-gene CDC in sexual as compared to asexual species indicating more effective selection on CUB in asexual species.

## Discussion

It has become established consensus among evolutionary biologists that sex and recombination increase the effectiveness of purifying selection, based on theoretical considerations and empirical evidence derived from a multiplicity of studies^12^. Our results do not match these studies: we find no evidence for accumulation of deleterious mutations in asexual hexapod species. Overall, asexual and sexual lineages neither differed in nonsynonymous site divergences corrected for background substitution rates (dN/dS), in synonymous substitution rates (dS) potentially influencing dN/dS ratios, in the ‘deleteriousness’ of nonsynonymous mutations nor in selection acting on CUB (CDC). Comparisons between sexual and asexual lineages within each group differed for several hexapod groups which may hint at group-specific differences in the consequences of asexuality on effectiveness of purifying selection. However, here, we refrain from drawing conclusions based on the within-group comparisons because the taxon sampling of 1KITE did not allow for analysing multiple within-group replicates but restrict the discussion to the overall result of more effective selection being absent in our data of sexual hexapods: which (non-mutually exclusive) mechanisms might be responsible for the discrepancy between this finding and the established consensus?

First, analyses of purifying selection acting at nonsynonymous sites and on their ‘deleteriousness’ may have been affected due to data limitations. dN/dS ratio analyses and ancestral state reconstructions rely on branch length estimates (see Methods). Due to the limited nature of the taxon sampling of the 1KITE data set for this study, the asexual and sexual species analysed are most likely not sister species, but rather more distantly related^46,59^. Therefore, the loss of sex did not occur with the split of the sex-asex species pair as present in the given phylogenetic tree here, such that evolution over some fraction of the asexual branch was likely sexual. If mutations occuring in asexual lineages did not accumulate at greatly increased rates compared with sexuals, a change in deleterious mutation accumulation along with the transition to asexuality might be masked by the rates that occured in the sexual fraction of the branch. Thus, the power to detect an effect of reproductive mode on the effectiveness of purifying selection might be low. Further, the orthologue search among phylogenetically distantly related hexapod groups and the stringent control for false positive orthologues and loci under positive selection resulted in a rather small orthologue set for analysis (99 orthologue loci). This biases the analyses towards strongly conserved loci and excludes recently evolved orthologues which might differ in accumulation of deleterious mutations between reproductive modes. Also, the within hexapod group comparisons between reproductive modes for CDC did not resemble those for dN/dS; in the case of Zygentoma they even opposed them (see Fig. 2a,b; Table 1). This is surprising, given that translational selection acting at synonymous sites is assumed to be weak and effective purging of synonymous mutations likely occurs at lower rates as compared to that of nonsynonymous mutations. Hence, within hexapod group comparisons of purifying selection acting at nonsynonymous sites should not oppose those at synonymous sites.

Second, the analysed asexual lineages may have lost sex too recently to have fixed enough deleterious mutations to be detected. This was for example also assumed to be responsible for absence of deleterious mutation accumulation observed in whole transcriptome data of fissiparous *Lineus* ribbon worms and four aphid species^14,18^. In fact, the occurrence of abundant males in some locally restricted populations of *N. phytophila*, *E. californica*, and *L. bostrychophila* analysed in this study is in line with a rather recent loss of sex^18^. Additionally, interference of rare, furtive, or cryptic sex with asexual genome evolution may explain the observed absence of less effective selection in asexual hexapods because rare events of sex are assumed to be sufficient to compensate for predicted consequences of asexuality^60^.

A third reason for the absence of deleterious mutation accumulation in the analysed data of asexual hexapod lineages may be effective homogenising mechanisms. Gene conversion and DNA repair have been shown to maintain DNA integrity, e.g. within the human Y chromosome, higher plant chloroplasts and animal mitochondria^61–63^. If homogenising mechanisms play a role in the analysed species remains to be investigated.

Fourth, besides reproductive mode, population size acts as a major determinant of effectiveness of selection with the speed of mutation accumulation being inversely related to population size, as shown by modelling approaches^9,64,65^. Large population sizes have been suggested to maintain effective purifying selection in asexual organisms, e.g. in oribatid mites and polyphagous pest insects, such as scale insects^15,66,67^. Further, large population sizes have been shown to increase the effectiveness of selection acting on CUB in a variety of animal species and on nonsynonymous mutations with large deleterious effects in *Caenorhabditis elegans*^68,69^. As information on population sizes of the analysed species is absent, any correlation between population sizes and the observed absence of more effective purifying selection acting at nonsynonymous and synonymous sites has to remain speculative.

In conclusion, our results do not support the established consensus of reduced effectiveness of purifying selection in asexual species, contrasting earlier analyses of few individual genes in a variety of animal and plant species. However, whether or not this is due to the limited nature of our data or due to non-mutually exclusive biological mechanisms has to remain elusive at this point. Future studies, hence, need not only to include large gene sets but also carefully selected closely related sexual and asexual focal lineages to study the effectiveness of purifying selection in asexual organisms in more detail.

## Supplementary information

Supplementary information

## Data Availability

Sequence data analysed in this study is available at NCBI GenBank under Accession numbers MH551269-MH551284, MH602437-MH602956, MH637812-MH638065 and MH799322-MH800185. Supplementary data is available for download from the digital repository DRYAD under 10.5061/dryad.5501rv4 (see Supplementary Archives S1–S4; Supplementary information).
