# Supplementary material for: No signal of deleterious mutation accumulation in conserved gene sequences of extant asexual hexapods
Source: Sci Rep. 2019 Mar 29;9:5338. doi: 10.1038/s41598-019-41821-x (PMC6441085; doi:10.1038/s41598-019-41821-x)
Supplement: Supplementary file 1 — Supplementary information [file 41598_2019_41821_MOESM1_ESM.pdf]

No signal of deleterious mutation accumulation in conserved gene sequences of extant asexual hexapods

Alexander Brandt, Jens Bast, Stefan Scheu, Karen Meusemann, Alexander Donath, Kai Schütte, Ryuichiro Machida, Ken Kraaijeveld

Supplementary information

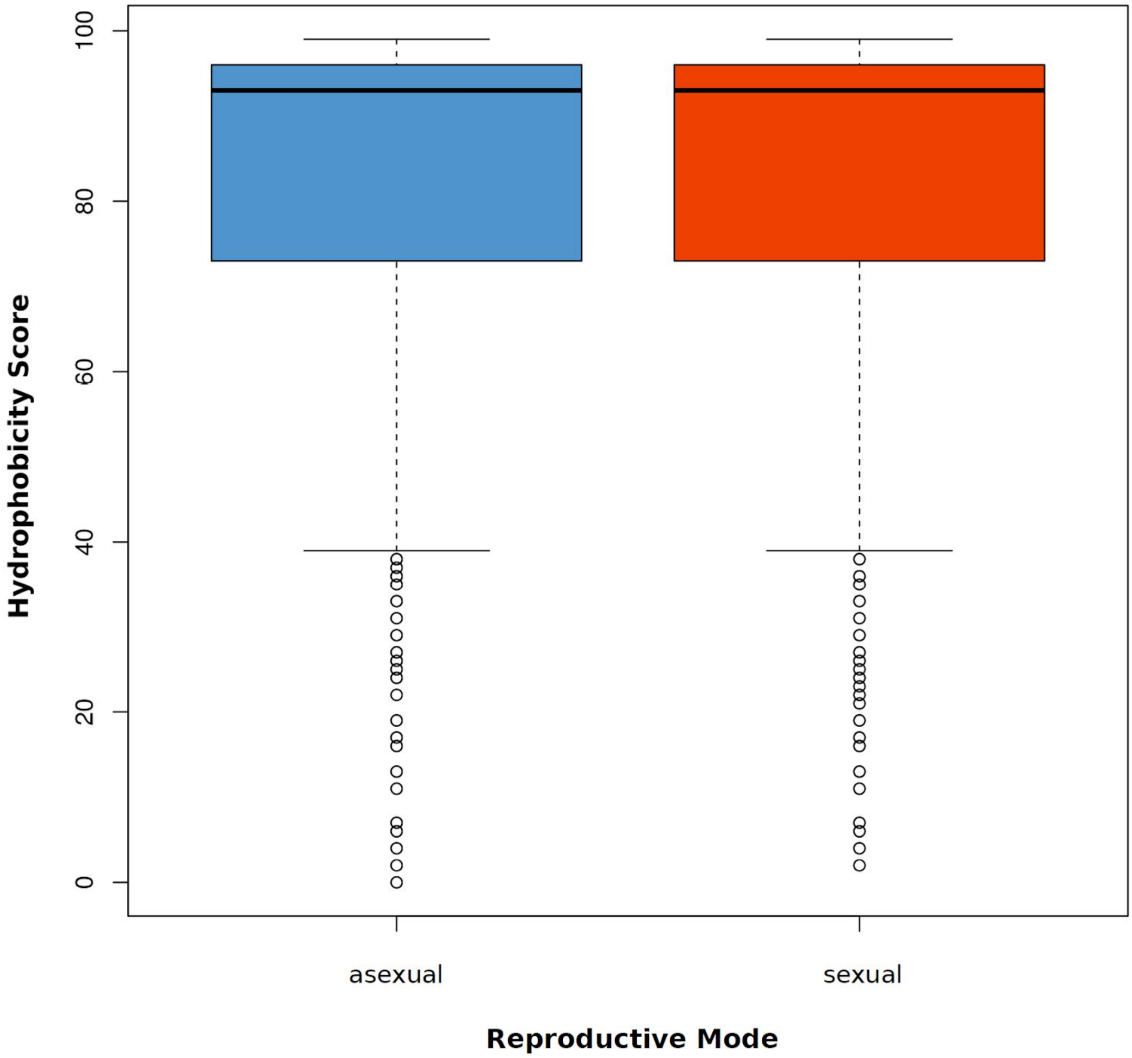

Supplementary Figure S1: Hydrophobicity scores at asexual and sexual terminal branches. The boxplots show Hydrophobicity Scores (HS) of eight asexual (blue) and eight sexual (red)

terminal branches for 73 nuclear orthologous genes shared among the 16 hexapod species and the outgroup *Xibalbanus* cf. *tulumensis* (involving 3,841 and 3,172 HS of asexual and sexual terminal branches, respectively). HS indicates the 'deleteriousness' of a nonsynonymous mutation by measuring the strength in hydrophobicity change from ancestral to replacement amino acid. The lower the HS the stronger is the change in hydrophobicity from ancestral to replacement amino acid and, hence, the deleteriousness of the underlying nonsynonymous mutation. There was no difference in hydrophobicity changes between reproductive modes (z = -0.152; P = 0.879; GLMM). Whiskers correspond to 1.5 times the interquartile range.

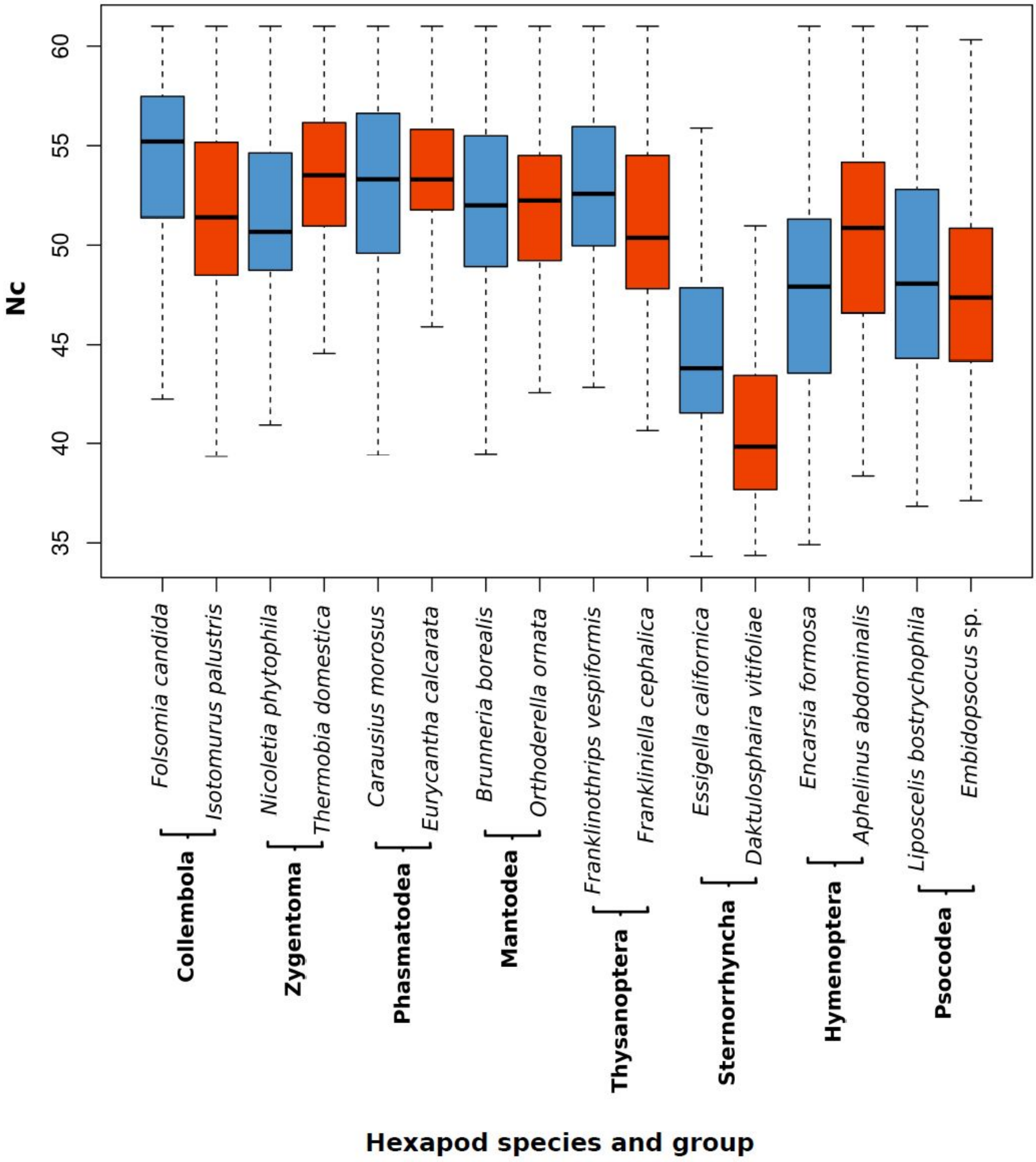

Supplementary Figure S2: Effective Numbers of Codons (Nc) of 16 hexapod species. The boxplots show the distributions of Nc of 99 orthologue loci for eight asexual (obligate asexual species present in the 1kite transcriptome data set; blue boxes) and eight sexual (red boxes) hexapod species, covering eight hexapod groups. For better representation, the ordinate is restricted to between 34 and 60; outliers outside of 1.5 times the interquartile range (whiskers) are excluded. Nc specifies the deviation of observed codon usage from

equal usage of all codons ranging from 20 (each amino acid is encoded by one codon only; strong CUB) to 61 (equal use of all possible codons; no CUB).

Supplementary Table S1: Additional information on sequence data analysed in this study

Species	First describer	Transcriptome assembly previously published in:	BioProject Accession of already published transcriptomes	BioSample Accession of species samples used in this study
Aphelinus abdominalis	Dalman, 1820	Peters *et al.*, 2018 [1]	PRJNA252351	SAMN02870213
Brunneria borealis	Scudder, 1896	not applicable		SAMN03339328
Carausius morosus	Sinety, 1901	not applicable		SAMN04005132
Daktulosphaira vitifoliae	Fitch, 1855	not applicable		SAMN03331961
Embidopsocus sp.	Genus by Hagen, 1866	not applicable		SAMN03331967
Encarsia formosa	Gahan, 1924	Peters *et al.*, 2018 [1]	PRJNA252167	SAMN02870290
Essigella californica	Essig, 1909	Misof *et al.*, 2014 [2]	PRJNA219554	SAMN02047099
Eurycantha calcarata	Lucas, 1869	not applicable		SAMN04005157
Folsomia candida	Willem, 1902	Misof *et al.*, 2014 [2]	PRJNA219557	SAMN02047120
Frankliniella cephalica	Crawford, 1910	Misof *et al.*, 2014 [2]	PRJNA219559	SAMN02047110
Franklinothrips vespiformis	Crawford, 1909	not applicable		SAMN03331968
Isotomurus palustris	Müller, 1776	not applicable		SAMN03142432
Liposcelis bostrychophila	Badonnel, 1931	Misof *et al.*, 2014 [2]	PRJNA219573	SAMN02047187

Nicoletia phytophila	Gervais, 1844	not applicable		SAMN03142452
Orthoderella ornata	Giglio-Tos, 1897	not applicable		SAMN04005206
Thermobia domestica	Packard, 1873	Misof *et al.*, 2014 [2]	PRJNA219608	SAMN02047119
Xibalbanus cf. *tulumensis*	Yager, 1987	von Reumont *et al.*, 2012 [3]	PRJNA66987	not available

Supplementary Table S2: GenBank Accession numbers of sequences (Open Reading Frames; ORFs) analysed in this study sorted by OMA orthologue group IDs. 70 orthologue groups include sequences of *X*. cf. *tulumensis* (used for analyses of 'deleteriousness' of nonsynonymous mutations; 17 sequences per orthologue group). For three orthologue groups, sequences of *X*. cf. *tulumensis* remained unchanged after ORF extraction and represent duplicates of sequences of the published transcriptome [3].

OMA orthologue group ID	GenBank Accession	Number of sequences per orthologoue group	Remarks
9945	MH638049 - MH638065	17	
9834	MH638032 - MH638048	17	
9690	MH638015 - MH638031	17	
9567	MH637998 - MH638014	17	
9559	MH637981 - MH637997	17	
9512	MH637964 - MH637980	17	
9320	MH637947 - MH637963	17	

9266	MH637930 - MH637946	17	
9067	MH637913 - MH637929	17	
9051	MH637896 - MH637912	17	
8832	MH637879 - MH637895	17	
8688	MH637862 - MH637878	17	
8442	MH637845 - MH637861	17	
8063	MH637828 - MH637844	17	
7631	MH637812 - MH637827	16	
7586	MH800169 - MH800185	17	
7514	MH800152 - MH800168	17	
7342	MH800135 - MH800151	17	
7199	MH800118 - MH800134	17	
6996	MH800102 - MH800117	16	
6954	MH800085 - MH800101	17	
6755	MH800068 - MH800084	17	
6720	MH800051 - MH800067	17	
6343	MH800035 - MH800050	16	OG6343_X_tulumensis_s is identical to TSA Accession JL198982 (von Reumont *et al.*, 2012) [3].
6321	MH800018 - MH800034	17	
6087	MH800001 - MH800017	17	

6076	MH799984 - MH800000	17	
5435	MH799968 - MH799983	16	
5260	MH799951 - MH799967	17	
5220	MH799934 - MH799950	17	
4377	MH799918 - MH799933	16	
4250	MH799902 - MH799917	16	OG4250_X_tulumensis_s is identical to TSA Accession JL138502 (von Reumont *et al.*, 2012) [3].
4143	MH799885 - MH799901	17	
4101	MH799869 - MH799884	16	
3827	MH799853 - MH799868	16	
3814	MH799836 - MH799852	17	
3745	MH799820 - MH799835	16	
3632	MH799803 - MH799819	17	
3464	MH799786 - MH799802	17	
3341	MH799770 - MH799785	16	
3278	MH799753 - MH799769	17	
2885	MH799737 - MH799752	16	
2874	MH799721 - MH799736	16	
2845	MH799704 - MH799720	17	
2780	MH799688 - MH799703	16	

2453	MH799671 - MH799687	17	
2434	MH799655 - MH799670	16	
2366	MH799638 - MH799654	17	
2249	MH799622 - MH799637	16	
1942	MH799605 - MH799621	17	
1925	MH799589 - MH799604	16	
1874	MH799573 - MH799588	16	
1858	MH799556 - MH799572	17	
1815	MH799539 - MH799555	17	
1641	MH799523 - MH799538	16	
1518	MH799507 - MH799522	16	OG1518_X_tulumensis_s is identical to TSA Accession JL174507 (von Reumont *et al.*, 2012) [3].
1499	MH799490 - MH799506	17	
1031	MH799474 - MH799489	16	
375	MH551269 - MH551284, MH799473	17	
17763	MH799456 - MH799472	17	
17620	MH799439 - MH799455	17	
17527	MH799423 - MH799438	16	
16696	MH799406 - MH799422	17	

16029	MH799390 - MH799405	16
15828	MH799373 - MH799389	17
15479	MH799356 - MH799372	17
15365	MH799339 - MH799355	17
14989	MH799322 - MH799338	17
14697	MH602941 - MH602956	16
14338	MH602924 - MH602940	17
14182	MH602907 - MH602923	17
14102	MH602890 - MH602906	17
13754	MH602874 - MH602889	16
13719	MH602857 - MH602873	17
13695	MH602840 - MH602856	17
13624	MH602823 - MH602839	17
13609	MH602806 - MH602822	17
12963	MH602790 - MH602805	16
12890	MH602774 - MH602789	16
12725	MH602758 - MH602773	16
12223	MH602742 - MH602757	16
11984	MH602725 - MH602741	17

11941	MH602708 - MH602724	17
11819	MH602691 - MH602707	17
11738	MH602674 - MH602690	17
11485	MH602657 - MH602673	17
11151	MH602641 - MH602656	16
10970	MH602624 - MH602640	17
10837	MH602607 - MH602623	17
10733	MH602590 - MH602606	17
10625	MH602573 - MH602589	17
10598	MH602556 - MH602572	17
10571	MH602539 - MH602555	17
10467	MH602522 - MH602538	17
10418	MH602505 - MH602521	17
10379	MH602488 - MH602504	17
10272	MH602471 - MH602487	17
10257	MH602454 - MH602470	17
10011	MH602437 - MH602453	17

List of archives available for download from DRYAD under doi:10.5061/dryad.5501rv4 (Supplementary Archives S1-S4):

Supplementary Archive S1 includes:

- curated codon alignments of 99 orthologue groups (OGs; FASTA-format) used for analyses of nonsynonymous and synonymous mutation accumulation (see Methods). Each alignment filename corresponds to the respective OG (see Supplementary Table S2).
- CodeML control file (PAML version 4.9) used for analyses of nonsynonymous mutation accumulation
- Python script for passing fixed species trees with loci-specific branch lengths and curated codon alignments to CodeML for analyses of nonsynonymous mutation accumulation (detailed information is given in the script)

Supplementary Archive S2 includes:

- curated codon alignments of 73 orthologue groups (OGs; FASTA-format) used for analyses of 'deleteriousness' of nonsynonymous mutations (see Methods). Each alignment filename corresponds to the respective OG (see Supplementary Table S2).
- CodeML control file (PAML version 4.9) used for prediction of ancestral amino acid states
- Python script for passing fixed species trees with loci-specific branch lengths and curated codon alignments to CodeML for prediction of ancestral amino acid states (detailed information is given in the script)

Supplementary Archive S3 includes:

- R-script for statistical analyses of Hydrophobicity Scores (‘deleteriousness’ of nonsynonymous mutations; detailed information is given in the R-script)

Supplementary Archive S4 includes:

- codon alignments of 286 orthologue groups resulting from initial orthology inference with OMA
- codon alignments of 153 orthologue groups resulting from searching the 286 initial orthologue groups against a precompiled set of orthologues with HaMStR (see Methods)

Supplementary References

1. Peters, R. S. *et al.* Transcriptome sequence-based phylogeny of chalcidoid wasps (Hymenoptera: Chalcidoidea) reveals a history of rapid radiations, convergence, and evolutionary success. *Mol. Phylogenet. Evol.* **120,** 286–296 (2018).
2. Misof, B. *et al.* Phylogenomics resolves the timing and pattern of insect evolution. *Science* **346,** 763–767 (2014).
3. von Reumont, B. M. *et al.* Pancrustacean phylogeny in the light of new phylogenomic data: support for Remipedia as the possible sister group of Hexapoda. *Mol. Biol. Evol.* **29,** 1031–1045 (2012).
